# Subjectively intense odor does not affect dream emotions during rapid eye movement sleep

**DOI:** 10.1038/s41598-023-37151-8

**Published:** 2023-06-27

**Authors:** Satomi Okabe, Takashi Abe

**Affiliations:** 1 International Institute for Integrative Sleep Medicine (WPI-IIIS), University of Tsukuba, 1-1-1 Tennodai, Tsukuba, Ibaraki 305-8575 Japan; 2 Graduate School of Comprehensive Human Sciences, University of Tsukuba, 1-1-1 Tennodai, Tsukuba, Ibaraki 305-8575 Japan; 3 Research Fellowship for Young Scientists (PD), Japan Society for the Promotion of Science, 5-3-1 Koujimachi, Chiyoda, Tokyo, 102-0083 Japan; 4 Department of sleep-wake disorders, National Center of Neurology and Psychiatry, 4-1-1 Ogawa-Higashi, Kodaira, Tokyo, 187-8551 Japan

**Keywords:** Cognitive neuroscience, Emotion, Sensory processing, Neuroscience, Psychology

## Abstract

Dreams experienced during rapid eye movement (REM) sleep have emotional features. Intervention methods for dream affectivity have recently garnered interest; we previously demonstrated that negative dreams were induced during REM sleep by exposure to favorable or familiar odors. However, the underlying mechanisms behind this phenomenon remain unclear. Thus, to address this gap, we investigated whether more intense odors could induce negative dreams, as odors tend to be perceived as more intense when they are preferred or familiar. Contrary to our hypothesis, the results of our study indicated that subjective intense odors did not induce negative dreams. We initially anticipated stronger odors to have a greater impact on dream emotionality, as they stimulate the brain more intensely. Notably, during arousal, weak odors tended to evoke a more potent olfactory response, while strong odors tended to produce a weaker response. To investigate whether this difference influenced the effects on dreams, we compared the respiratory activities of the strongly and weakly perceived odor groups; however, no significant differences were observed. Our findings suggest that subjectively perceived strong odors are unlikely to affect dream emotionality and may be processed differently than favorable or familiar odors.

## Introduction

Dreams during rapid eye movement (REM) sleep are characterized by emotional features^1,2^. The importance of studying dream emotionality has been emphasized owing to its clinical significance. Nightmares, which are dreams with excessively negative emotions, are associated with several mental illnesses, including depression^3^, suicide attempts^4,5^, insomnia^6^, and post-traumatic stress disorder^7^. Emotionality is an important variable for describing and understanding dreams as subjective experiences from a basic research perspective.

Odor is a useful stimulus for examining the processing of information during sleep^8^. Odors without trigeminal components^9,10^ and mild trigeminal odors^11^ cause neither an increase in the arousal levels nor notice of odor presentation; moreover, several studies have reported odors to be processed during sleep^8,12–14^. However, during awakening, odor perception has highly variable individual differences^15^ that should be considered when implementing odors in sleep research.

Our previous studies^16,17^ revealed that specific individual variations in odor perception are related to the effects of olfactory presentation during REM sleep on dream emotionality. First, we investigated whether the effects of odor on dream emotionality were associated with individual variations in odor preference and found that only preferred odors induced more negative dream emotions^16^. In a subsequent study, we found that familiar odors also induced negative dreams^17^. Odor preference and familiarity are correlated with subjective intensity ratings^18–20^. Since a more intense odor (stronger perceptual experience) generally results in stronger brain activity^15^, the findings of our previous studies^16,17^ could be attributed to odor preference and familiarity, reflecting how intensely the odor is perceived.

Moreover, odor sensitivity should also be investigated because it could be related to the perception of the odor intensity. The sensitivity and subjective intensity are very closely related perceptive characteristics. For example, some individuals with high sensitivities can perceive even low concentrations of chemical compounds, whereas others with low sensitivities can only perceive high concentrations. Several previous studies have indicated that repeated exposure to odors could increase sensitivity^21,22^, as well as subjective intensity^23^. However, the literature also indicated that the sensitivity and subjective intensity are independent indices and are not same or correspond to each other^24,25^. Repeated exposure to isobornyl acetate and citralva odors did not alter the participants’ subjective intensity ratings; however, their sensitivities to the odor improved^26^. Thus, it is important to examine not only the subjective intensity, but also sensitivity.

Therefore, the current study aimed to compare the effects of olfactory stimulation during REM sleep on dream emotionality between groups that perceived the odor as subjectively intense or weak. A sensitivity test was also conducted, and the correlation between sensitivity and the effect of odor on dream emotionality was investigated. Phenylethyl alcohol (rose-like odor; PEA) was used for olfactory stimulation, because it does not have trigeminal components^27,28^. PEA has been previously used in many sleep studies^8,13,16,17,29^. We hypothesized that the dreams of participants who perceived the odor as intense would be more emotionally negative by odor presentation and that high sensitivity would correlate with a stronger effect, thereby resulting in more negative dreams.

## Methods

This study was conducted at the University of Tsukuba (Tsukuba, Ibaraki, Japan), approved by the Clinical Research Ethics Review Committee of the University of Tsukuba Hospital (ethics review number: R01-141), and registered in the University Hospital Medical Information Network Clinical Trials Registry (UMIN-CTR; study number: UMIN000039772). This study was also performed in accordance with the Declaration of Helsinki. All the participants provided written informed consent before participation in the study. The methods used in this study are consistent with those used in our previous studies^16,17^. Before starting the experiment, the participants were only informed that odor presentation would be conducted during sleep and not about the nature of the odor or when it would be presented to avoid subjective bias attributed to knowledge of odor presentation during sleep.

### Participants

To determine the target sample size, sample size calculation using a two-tailed test, with α = 0.05 and power = 0.8, was performed based on the data of our previous studies assuming that the effect size would be the same as that in the previous study. The target sample sizes were 14 participants per group for an effect size of the 2018 results^16^ (d = 1.12) and 9 participants for the effect size of the 2020 results^17^ (d = 1.43). We adopted the sample size of the 2018 study^16^ and added 6 additional participants to each group to account for discontinuation or withdrawal. Therefore, the target sample size was set at 20 participants per group, for a total of 40 participants.

First, the participants were recruited for screening. The inclusion criteria for screening participants were as follows: aged between 20 and 25 years, have no difficulty filling out Japanese-language documents, and were able to stay in the laboratory sleep chamber. The exclusion criteria were as follows: presently have or history of smell-taste disorders, presently have or history of sleep disorders, use drugs known to affect smell-taste functions and sleep, have claustrophobia, confirmed or possible pregnancy, are currently breastfeeding, presently have or history of illness with a potential for sudden change, and were determined to be unsuitable for other safety reasons. According to these criteria, young healthy adults (n = 284; mean age: 22.0 ± 1.4; 130 men and 154 women) were screened. All the participants provided written informed consent.

Second, screening was performed to determine the subjective intensity groups (i.e., intense or weak). The classification criteria for the subjective odor intensity of PEA were as follows: rating intensity of 1–3 for the weak group, and 6–9 for the intense group. Preference and familiarity were controlled by limiting these ranges inside the areas of the mean ± standard deviation (SD) of the distribution of previous data (preference: < 3 and > 6; familiarity: < 4 and > 7). Participants who passed the screening were fully informed of the description of the experiment and signed consent forms before the experiment.

Initially, the weak and intense groups were determined to be outside the areas of the mean ± SD of the distribution of intensity rating from our previous data^16,17^ (initial criteria: weak < 3, strong > 8). However, since the distribution of subjective intensity was different between the previous and present data (previous: 5.4 ± 2.1; present: 4.3 ± 2.1), we could not select a sufficient number of participants based on the original criteria. Thus, we had to change the criteria for participant recruitment before completing the experiment.

To summarize, a total of 40 participants took part in the sleep experiment (mean age: 21.6 ± 1.2 years; 20 men and 20 women; weak group: n = 20; strong group: n = 20). Of these, 7 participants (mean age: 21.9 ± 0.3 years; 4 men and 3 women) were excluded from subsequent analyses according to our previous studies^16,17^: three noticed the stimulus presentation (dreams might be affected), and four reported that their REM sleep did not continue for sufficient lengths (protocols could not be completed). Thus, this study analyzed the data of the remaining 33 participants (mean age: 21.5 ± 1.3 years; 16 men and 17 women; weak group: n = 17; strong group: n = 16).

### Procedures

In the screening session, the participants smelled the PEA, citral, and eugenol samples by sniffing porous beads perfumed with 10% PEA (solvent: triethyl citrate) in a vial and answered a questionnaire consisting of their subjective impressions of the odors using adjective scales. The scales ranged from 1 to 9, including intensity (1 [weak] to 9 [strong]), familiarity (1 [unfamiliar] to 9 [familiar]), and preference (1 [unpreferred] to 9 [preferred]) (Table S1); 10% citral and 10% eugenol (solvent: triethyl citrate) samples were used as dummies to prevent participants from noticing or guessing the experimental stimuli; thus, the ratings of these odors were not used for participant selection. The researcher instructed that the participants could sniff the odor sample any number of times while they answered the questionnaire. Only the ratings of the PEA sample were used for participant selection.

In the sleep experiment session, the participants arrived at the laboratory 2 h before their usual bedtimes. They subsequently completed the State-Trait Anxiety Inventory^30,31^, Global Vigor and Affect^32^, and other visual analog scale items generated for the study (Table S2) to evaluate their pre-sleep conditions. After polysomnography, the participants went to the sleep chamber. The lights were turned off at their usual bedtimes (12:11 a.m. ± 1:05), and after switching off the lights, the participants were allowed to sleep. Adaptation nights were taken place for all the participants to suppress the first night effect^33^.

PEA, as the experimental stimulus, and distilled water (DW), as the control stimulus, were presented in a counterbalanced order during the second and subsequent REM periods. The participants were woken up 1 min after the end of odor presentation and asked to report and rate their dreams. If the participant reported that they did not have dreams or if REM sleep did not continue for 10 min, the next REM period was provided for dream collection. After dream collection in the experimental and control conditions, the participants were allowed to sleep until their usual waking times.

Participants evaluated their post-sleep conditions the next morning after the experiment using the same questionnaire as that before sleeping. Subsequently, the participants underwent an examination to test their sensitivities to PEA, which was thought to be related to the perceived intensity of the odor and may correlate with its effects. For this examination, a standard odor set for panel selection (Daiichi Yakuhin Sangyo) was used. This set was constructed using nine concentrations of five chemical compounds, including PEA. In this study, only PEA was used. At the end of the experiment, the participants were informed about the timing of odor presentation and inclusion criteria for the subjective intensity of the PEA odor. The participants were also asked whether they noticed the odor stimulation.

### Sleep recordings

Polysomnography was performed during the adaptation and experimental nights. Six electroencephalograms (F3-A2, F4-A1, C3-A2, C4-A1, O1-A2, and O2-A1), two electrooculograms (left and right), and two submental electromyograms (bipolar leads) were performed according to the American Academy of Sleep Medicine (AASM) criteria^34^. Respiration was recorded using a thermistor (AP-C025, Miyuki Giken. Co., Ltd., Tokyo, Japan). Polymate Pro MP6100 (Miyuki Giken) Co., Ltd., Tokyo, Japan) was used for measurement. Sleep stages were scored according to the AASM criteria^34^.

### REM awakenings, self-ratings, and self-reports of dreams

The participants were woken up twice (control and odor conditions) during nocturnal sleep for reporting their dreams. First, they evaluated their dreams using the Emotional Tone^8^ and Dream Property (DP)^35,36^ Scales. Emotional tone^8^ is calculated according to subjectively evaluated positive and negative dream emotion scores (0 = none, 1 = mild, 2 = moderate, and 3 = strong). The negative score was subtracted from the positive score, and the resulting value was defined as the emotional tone. The DP Scale^35,36^ is a structured questionnaire with four dream property factors: emotionality, rationality, activity, and impression. This 7-point scale (1–7) contains 15 items to determine the dream characteristics. The scores for each factor are calculated by summing the scores of the items within each factor. The emotionality factor included four items; hence, the minimum score is 4 (very negative dreams), and the maximum score is 28 (very positive dreams).

After the self-rating of dreams, the participants were asked, “What was on your mind before I woke you up?” After a pause during the reporting, the question, “Was there anything else?” was asked as follow-up three times.

During the sleep experiment, conversations between the participants and researcher were minimized. When the participants were in the sleep chamber, all communications were carried out through an intercom, and no face-to-face contact occurred. Moreover, the dream interviews were strictly structured. However, the researcher was not blinded to the order of the two experimental conditions (control and odor) or participant groups (weak and strong).

### Olfactory stimulation

**Figure 1:**
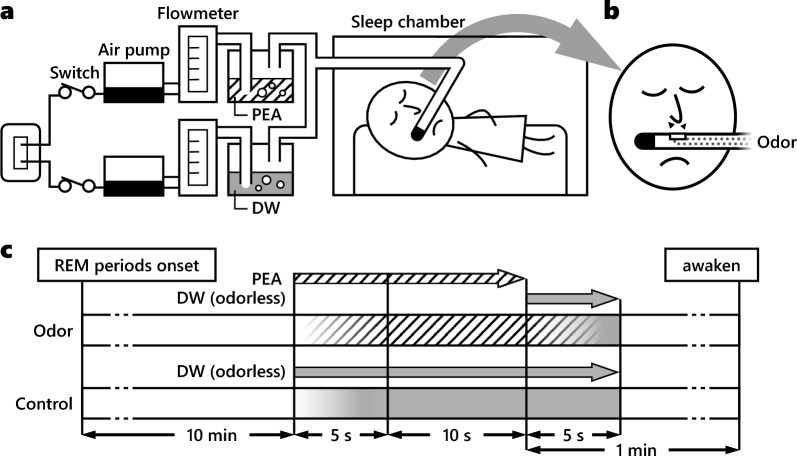
Olfactory stimulation device and odor presentation protocol. The device allowed the presentation of evaporated phenylethyl alcohol (PEA) and distilled water (DW) in an airstream, according to the protocol. (**a**) Device structure: the airstream is generated by an air pump and regulated by a flowmeter. PEA and DW evaporated upon bubbling and passed through a Teflon tube, 350 cm in length. This device was located outside the sleep chamber; therefore, the noise and vibration of the device did not disturb sleep. (**b**) Attachment of the olfactory device to the participants: the stimulation was presented through the small orifice of the tube attached just below the nostrils of the participants. (**c**) The protocol: stimulation was initiated 10 min after the onset of the rapid eye movement (REM) sleep period. The airflow required 5 s to reach the participants from the device. PEA and DW airflows were presented for 10 s under odor and air conditions, respectively. After stimulation of the odor condition, the DW airflow was released for 5 s to remove air from the tube. The 5-s DW airflow was also applied to the control condition to match the protocol for both conditions. (**a**) and (**b**) are reworked from our previous papers^16,17^, and reuse is permitted by the publisher.

An olfactory stimulation device was constructed for this study as shown in Fig. 1a,b. PEA and DW evaporated upon bubbling through a continuous airstream, each flowing at 4 L/min, through a Teflon tube. An air pump (HIBLOW CD-8S; TECHNO TAKATSUKI) was used to regulate airflow using a flowmeter with a precision needle valve (model 1250 series; KOFLOC).

The protocol for the stimulus presentation is shown in Fig. 1c. Stimulation began 10 min after the onset of the REM sleep period. In this study, REM sleep was scored in real time; therefore, REM sleep onset was defined as the point at which three features of REM sleep (low-voltage electroencephalogram record, muscle atonia, and REMs) were observed. The airflow from the device took 5 s to reach the participants. Each PEA airflow during the odor condition and DW airflow during the control condition was presented for 10 s. After the stimulation, a 5-s odorless airflow (DW airflow) was added to clear the previous air. Therefore, the effective stimulation lasted for 10 s, whereas pre- and post-stimulation lasted for 5 s each (Fig. 1c). The switch-controlled device was installed outside the sleep chamber to minimize sleep disturbances caused by noise, light, or mechanical vibrations.

### Post-hoc analysis for respiration

**Figure 2:**
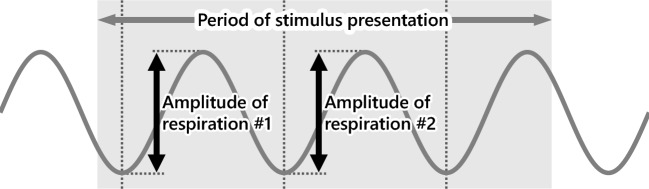
Post-hoc analysis for respiration. Black arrow: amplitude of respiration. Gray arrow: the period of stimulus presentation (10 s). Gray solid line: example of a respiration waveform.

The findings of this study demonstrate that subjective intensity has no influence on dreams, as evidenced in the results section. This may be attributed to a potentially diminished sniffing response in the intense group. Therefore, we conducted an analysis to examine this possibility. The peak-to-peak amplitudes of the waveforms were measured. For instances in which multiple respiratory waveforms were present within the interval, the amplitudes were computed as averages, yielding individual data for both the odor and control conditions. The average values were compared for each group (Fig. 2). After excluding three participants due to the absence of stimulation and two participants due to inadequate measurement data, this analysis included data from 28 participants.

### Statistical analysis

Pearson’s correlation coefficients were used to explore the correlation between the ratings of subjective impressions of PEA at screening, as well as that between subjective intensity and sensitivity in the experiment. A two-tailed t-test was used to compare the backgrounds, pre- and post-sleep conditions, and experimental conditions between the groups. Analysis of variance (ANOVA) was used to examine the effects of odor and subjective intensity ratings (groups) on dream emotionality. All the analyses were performed using IBM SPSS Statistics for Windows, version 27 (IBM Corp., Armonk, N.Y., USA). The alpha level was set at 0.05. Sample size calculation was performed using G* Power 3.1.9.4.^37,38^. Bayesian ANOVA was performed using JASP 0.17.2.1.^39^.

## Results

The ratings of subjective impressions of PEA from the screening session (n = 284) demonstrated significant correlations between subjective intensity and familiarity; a more intense perceived odor was associated with more familiarity (r = 0.183; p < 0.01). Subjective intensity also correlated with preference, such that unpreferred odors tended to be perceived as more intense (r = -0.147; p = 0.01). The other correlation coefficients are listed in Table 1.

**Table 1:** Odor rating score and correlation with odor intensity.

	Mean	(SD)	Correlation coefficients (r) with odor intensity	p
Cheap-expensive	4.79	(1.75)	0.094	0.11
Warm-cold	4.22	(1.95)	0.099	0.10
Masculine-feminine	6.26	(1.90)	0.022	0.71
Calm-activating	3.17	(1.62)	0.357*	0.00
Weak-intense	4.28	(2.05)	–	–
Have never smelled-have smelled	7.04	(1.86)	0.187*	0.00
Unfamiliar-familiar	6.26	(1.99)	0.183*	0.00
Nostalgic-novel	3.96	(1.64)	0.047	0.43
Feel distant from-feel close to	5.81	(1.84)	0.008	0.90
Positive–negative	4.14	(1.95)	0.103	0.08
Unattractive-attractive	5.47	(2.11)	0.035	0.55
Pleasant-unpleasant	3.95	(1.78)	0.276*	0.00
Unpreferred-prefer	5.85	(1.81)	− 0.147*	0.01

Means and (standard deviations) are shown. Several indices were significantly correlated with odor intensity ratings. *p < 0.05.

After the screening, 40 participants were recruited for the sleep experiment. Even after the exclusion of data (see details in the participants part of the methods section), no significant differences were observed in the odor preference scores between the weak and strong groups (mean ± SD: 4.9 ± 1.2 and 4.1 ± 1.1, respectively; two-tailed t-test: t (31) = 1.88, p = 0.07) and familiarity scores (6.2 ± 0.8 and 6.3 ± 0.9, respectively; two-tailed t-test: t (31) = 0.25, p = 0.86). Therefore, the data from 33 participants were used in the subsequent analysis. Two-tailed t-tests between the groups revealed no significant differences in the age, preference, and familiarity with PEA at screening, pre- and post-sleep conditions, time of stimulation, and sensitivity to odor (Table 2). Chi-square test revealed that the proportion of sex also did not differ between the groups (χ^2^ (1) = 0.279; p = 0.60). No significant correlation was observed between the odor sensitivity and subjective intensity ratings (r = 0.167; p = 0.35).

**Table 2:** Comparison of the pre- and post- sleep conditions, sensitivity for odor, and time of stimulation between the groups.

	Weak		Intense		t	p
Age	21.5	(1.1)	21.5	(1.5)	0.06	0.95
Pre-sleep condition						
STAI						
State anxiety	58.82	(7.30)	61.19	(8.35)	0.84	0.41
Trait anxiety	51.76	(10.13)	54.69	(10.15)	0.80	0.43
GVA						
Vigor	57.91	(14.17)	51.84	(9.61)	1.39	0.18
Affect	69.96	(9.89)	73.04	(14.09)	0.62	0.54
VAS						
Energy	51.71	(19.04)	46.50	(13.09)	0.88	0.38
Tiredness	55.65	(16.86)	52.81	(17.03)	0.47	0.64
Worry about own health	51.18	(27.79)	57.75	(24.45)	0.70	0.49
Irritation	16.47	(19.47)	17.00	(16.52)	0.08	0.94
Motivation	47.65	(20.74)	50.13	(15.83)	0.37	0.71
Anxiety	35.71	(25.74)	35.25	(23.36)	0.05	0.96
Depression	30.65	(25.80)	25.44	(23.86)	0.58	0.56
Post-sleep condition						
STAI state anxiety	62.00	(5.59)	62.00	(5.48)	0.00	1.00
GVA						
Vigor	62.35	(14.29)	55.23	(14.05)	1.40	0.17
Affect	74.93	(7.88)	70.23	(7.64)	1.68	0.10
VAS						
Energy	46.82	(17.30)	47.75	(15.21)	0.16	0.88
Tiredness	36.94	(25.39)	42.06	(14.95)	0.68	0.50
Worry about own health	45.29	(30.68)	47.69	(23.08)	0.24	0.81
Irritation	14.41	(16.45)	17.88	(13.61)	0.64	0.53
Motivation	46.06	(20.31)	43.38	(10.08)	0.46	0.65
Anxiety	20.29	(23.37)	23.19	(16.14)	0.40	0.69
Depression	19.59	(23.36)	19.63	(16.44)	0.01	1.00
Time of stimulation						
Control condition	5:00:37	(1:48:30)	5:44:50	(1:40:50)	1.21	0.24
Odor condition	4:42:25	(1:39:20)	5:09:54	(1:40:22)	0.79	0.44
Sensitivity for odor	-5.24	(0.77)	-5.41	(0.92)	0.58	0.57

Means and (standard deviations) are shown. Significant differences between the groups were not observed. STAI: State-Trait Anxiety Inventory^30,31^, GVA: Global Vigor and Affect^32^, VAS: visual analog scale constructed for our study. The value of sensitivity for odor indicates 10^X^, and the range of X were − 3 (highest concentration) to − 7 (lowest concentration). In this study, the mean sensitivity was calculated using the X value and not the 10^X^ value, according to a previous study^58^.

**Figure 3:**
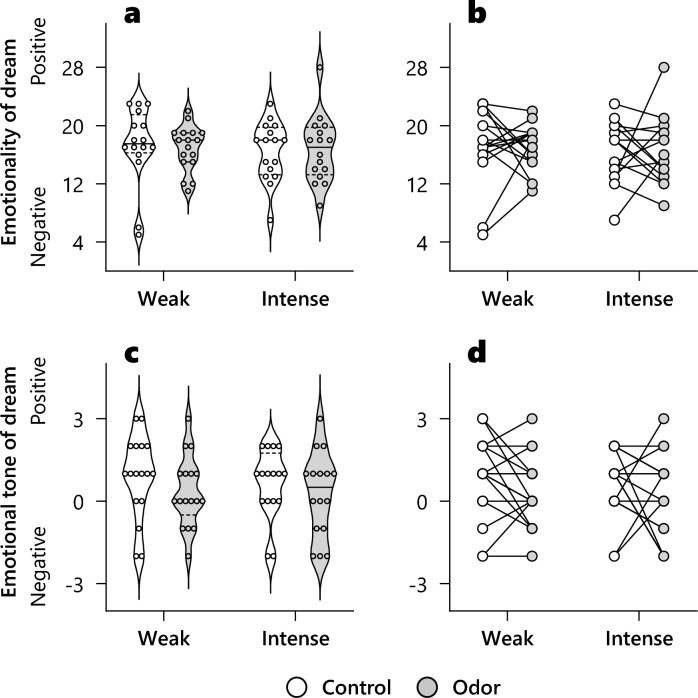
The effect of odor on the emotional tone and emotionality of dreams: comparison between individual differences on odor subjective intensity. (**a**,**c**) Violin plots. solid line: mean, dotted line: interquartile range. (**b**,**d**) Individual changes between conditions. White indicates odorless control condition. Gray is odor condition with PEA odor. Each small circle indicates individual data. There were no significant main effect and interaction.

We conducted two-factor repeated-measures ANOVA for the groups (weak and intense) and conditions (control and odor) concerning emotional tone^8^ (Fig. 3a,b) and emotionality of dreams^35,36^ (Fig. 3c,d). Both groups, conditions, and interactions had no significant effects. Other dream characteristics also demonstrated no significant effects (Table 3).

**Table 3:** Analysis of variance of the effect of odor on the dream rating.

	Weak		Intense		F		
	Control	Odor	Control	Odor	Group	Condition	Interaction
Emotional tone	0.88 (1.50)	0.35 (1.27)	0.63 (1.26)	0.25 (1.53)	0.01	2.74	0.21
Bizarreness of DPS	16.50 (5.66)	15.13 (5.75)	13.25 (7.28)	12.25 (6.24)	3.46	1.05	0.15
Emotionality of DPS	17.31 (5.30)	16.88 (3.22)	16.50 (4.07)	16.69 (4.57)	0.10	0.07	0.19
Impression of DPS	16.71 (4.87)	16.88 (4.96)	16.56 (4.11)	15.94 (4.70)	0.94	0.93	0.51
Activity of DPS	13.12 (3.14)	12.59 (3.71)	11.19 (3.76)	11.81 (3.19)	1.05	0.08	1.52

Means and (standard deviations) are shown. Degrees of freedom: 1; degrees of freedom for error: 29. No significant effects or interactions were observed. DPS: Dream property scale^35,36^.

We also conducted Bayesian repeated-measures ANOVA to reveal whether the data were inconclusive or indicated a null hypothesis. BF_01_ (the index providing the evidence in favor of the null hypothesis) of the group, condition, and interaction were 2.936, 3.997, and 11.721, respectively. On the other hand, BF_10_ (favor of the alternative hypothesis) were 0.341, 0.250, and 0.085 (group, condition, interaction, respectively). According to the criteria of Kass & Raftery^40^, BF_01_ provided, weak (group) and positive (condition and interaction) evidence in favor of the null hypothesis, while BF_10_ did not provide evidence for the alternative hypothesis.

**Figure 4:**
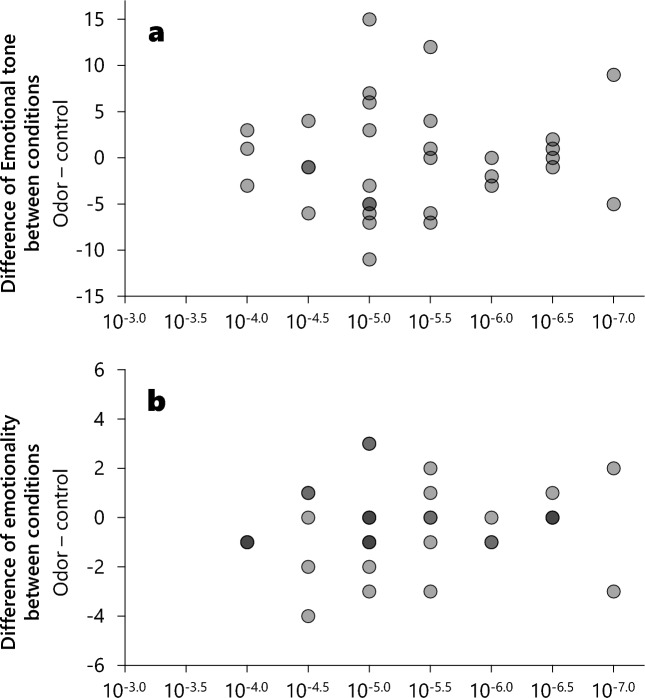
Correlation between odor sensitivity and the effects of odor on dreams. The gray points represent individual data, and the darker areas indicate overlapping data. The x-axis indicates sensitivity to odor: the lowest concentration that can be perceived by individual participants. The y-axis indicates the effects of odor on dreams: the difference in dream ratings between the odor and control conditions. (**a**) Emotional tone of dreams^8^. (**b**) Emotionality of dreams^35,36^. No significant correlations were identified on both scatter plots.

No significant correlations were identified between the effects of the odor (difference between odor and control condition on emotional tone^8^ and the emotionality of dreams^35,36^) and odor sensitivity (emotional tone: r = 0.133, p = 0.46; emotionality: r = 0.069, p = 0.71; Fig. 4).

**Figure 5:**
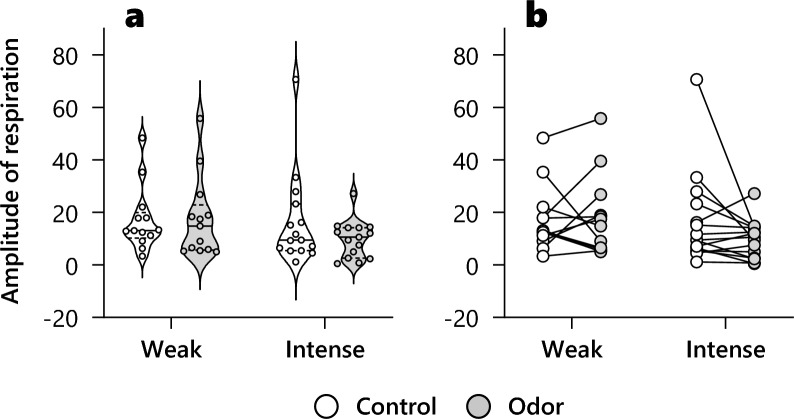
The effects of odor on amplitude of respiration (**a**) Violin plots. solid line: mean, dotted line: interquartile range. (**b**) Individual changes between conditions. White indicates odorless control condition. Gray is odor condition with PEA odor. Each small circle indicates individual data. There were no significant main effect and interaction.

In addition, we conducted two-factor repeated-measures ANOVA for the groups and conditions on respiration amplitude (Fig. 5). The groups, conditions, or interactions demonstrated no significant effects (group: F (1, 26) = 0.93, p = 0.34; condition: F (1, 26) = 1.22, p = 0.28; interaction: F (1, 26) = 1.60, p = 0.22).

## Discussion

In the present study, a subjectively intense odor had no effect on the dream emotions. Our previous studies revealed that preferred and familiar odors induced negative dreams^16,17^. Odor pleasantness (hedonic strength) and familiarity ratings correlated with the intensity ratings^18^; therefore, we hypothesized that a subjectively intense odor might induce negative dreams and that high sensitivity would correlate with a more negative odor, similar to preferred and familiar odors. However, this hypothesis was not supported.

In a previous study, we discovered that preferred and familiar odors induced negative dream emotions, whereas unpreferred and unfamiliar odors did not affect dreams^16,17^. During wakefulness, pleasant odors are inhaled more often than unpleasant odors^41^. This sniffing response also occurs during sleep^12^. Our previous study may explain this sniffing response during REM sleep. Specifically, pleasant odors (preferred and familiar) are likely to be strongly inhaled, resulting in greater amounts of odor information reaching the brain and having a stronger influence on dreams. In contrast, no difference was observed in the dream emotionality between the two groups in this study. During wakefulness, strong odors are weakly inhaled, whereas weak odors are strongly inhaled^42–44^. Currently, no evidence suggests whether this sniffing response occurs during sleep; however, if it does, the results of this study could be explained by the sniffing response. Therefore, the hypothesized correlation between the subjective intensity of odors and their effects on dreams could have been underestimated owing to the weak inhalation of a subjectively intense odor. However, the post-hoc analysis of the sniffing response revealed that the intense group did not demonstrate a weaker sniffing pattern, thus negating the hypothesis that the subjective intensity of the odor correlated with its effect on dreaming. Additionally, our findings suggest that the relationship between odor sensitivity and dreams is inconclusive, thereby supporting the hypothesis that favorable and familiar odors that are perceived as intense odors have a stronger effect on dreams.

The amygdala is part of the brain network that underlies the genesis of negative emotions^45^ and therefore plays an important role in the emergence of unpleasant dreams^46^. The olfactory tubercle, which is situated near the amygdala, receives direct input from the olfactory bulb, and is responsible for the preference for familiar odors^47^. In contrast, the evaluation of odor intensity primarily occurs in the orbitofrontal cortex^48^ through the thalamus. During sleep, the thalamus reduces sensory information transmission^49^. These differences in the processing pathways may account for variations in the results. Therefore, favorable and familiar odors induce unpleasant dreams^16,17^, whereas subjectively intense odors may not impact dreams. Furthermore, these odor patterns contribute to differences in sniffing responses during sleep, by which pleasant odors are inhaled more strongly while asleep^12^ and awake^41^, and strong odors are inhaled weakly during wakefulness^42–44^ but not during sleep.

This study aimed to compare the effects of odors between participants who perceived the odors as more intense and weaker, than as neutral in the subjective intensity rating distribution. A significant difference was observed in the odor intensity ratings between the groups. Therefore, we were able to compare the odor effect on the dreams between the groups based on the subjective intensity rating. However, as shown in the Participants section, the initial inclusion criteria of the odor intensity rating, as determined by the distribution of our previous data^16,17^, did not correspond to the current study. The reason for the variation in the distributions between the current and previous studies is unclear^16,17^. One of the differences between the previous and present studies was the presence of dummy odors during screening in the present study. However, this difference in the protocol could have had little effect on the PEA ratings. To suppress the effect of dummy odors on the PEA odor ratings, we performed PEA ratings first and dummies second and third. The influence of the coronavirus disease-2019 (COVID-19) pandemic should also be taken into consideration. Several studies have demonstrated COVID-19 to impair odor perception^50–52^. We excluded participants with smell-taste disorders, but not those with histories of COVID-19.

Our study has several limitations. In this study, the participants were limited to younger adults; therefore, the results cannot be generalized to other age groups. Olfactory perception declines with aging^53–55^. Therefore, when this study is applied to other age groups, the results could vary. In addition, we only used PEA odor as an experimental stimulus in this study. Thus, the effect obtained with the use of other types of odors is unclear. Several odors have general (not subject specific) effects on emotion^56^ and sleep^57^; thus, in the case of such odors, the general effects could confound to the effects selective to individual variation that we previously reported^16,17^.

In conclusion, this study indicates that individual variations in odor intensity do not affect dream emotionality, contrary to the hypothesis based on our previous study that studied individual variations in odor preference^16^ and familiarity^17^.

## Supplementary Information

Supplementary Tables.

## Data Availability

The datasets used during the current study are available from the corresponding author upon reasonable request.

## References

[1] Hobson JA, Pace-Schott EF, Stickgold R (2000). Dreaming and the brain: Toward a cognitive neuroscience of conscious states. Behav. Brain Sci..

[2] Nir Y, Tononi G (2010). Dreaming and the brain: From phenomenology to neurophysiology. Trends Cogn. Sci..

[3] Nakajima S (2014). Impact of frequency of nightmares comorbid with insomnia on depression in Japanese rural community residents: A cross-sectional study. Sleep Med..

[4] Agargun MY (2007). Nightmares, suicide attempts, and melancholic features in patients with unipolar major depression. J. Affect. Disord..

[5] Sjöström N, Hetta J, Waern M (2009). Persistent nightmares are associated with repeat suicide attempt. Psychiatry Res..

[6] Schredl M (2009). Nightmare frequency in patients with primary insomnia. Int. J. Dream Res..

[7] Rothbaum BO, Mellman TA (2001). Dreams and exposure therapy in PTSD. J. Trauma. Stress.

[8] Schredl M (2009). Information processing during sleep: the effect of olfactory stimuli on dream content and dream emotions. J. Sleep Res..

[9] Stuck BA (2007). Arousal responses to olfactory or trigeminal stimulation during sleep. Sleep.

[10] Carskadon MA, Herz RS (2004). Minimal olfactory perception during sleep: Why odor alarms will not work for humans. Sleep.

[11] Perl O (2016). Odors enhance slow-wave activity in non-rapid eye movement sleep. J. Neurophysiol..

[12] Arzi A (2012). Humans can learn new information during sleep. Nat. Neurosci..

[13] Rasch B, Buchel C, Gais S, Born J (2007). Odor cues during slow-wave sleep prompt declarative memory consolidation. Science.

[14] Bar E (2020). Local targeted memory reactivation in human sleep. Curr. Biol..

[15] Wilson DA, Stevenson RJ (2006). Learning to Smell: Olfactory Perception from Neurobiology to Behavior.

[16] Okabe S, Fukuda K, Mochizuki-Kawai H, Yamada K (2018). Favorite odor induces negative dream emotion during rapid eye movement sleep. Sleep Med..

[17] Okabe S, Hayashi M, Abe T, Fukuda K (2020). Presentation of familiar odor induces negative dream emotions during rapid eye movement (REM) sleep in healthy adolescents. Sleep Med..

[18] Distel H (1999). Perception of everyday odors-correlation between intensity, familiarity and strength of hedonic judgement. Chem. Senses.

[19] Ayabe-Kanamura S (1998). Differences in perception of everyday odors: A Japanese-German cross-cultural study. Chem. Senses.

[20] Distel H, Hudson R (2001). Judgement of odor intensity is influenced by subjects’ knowledge of the odor source. Chem. Senses.

[21] Yee KK, Wysocki CJ (2001). Odorant exposure increases olfactory sensitivity: Olfactory epithelium is implicated. Physiol. Behav..

[22] Wang L, Chen L, Jacob T (2004). Evidence for peripheral plasticity in human odour response: Olfactory plasticity in humans. J. Physiol..

[23] Stevenson RJ, Prescott J, Boakes RA (1995). The acquisition of taste properties by odors. Learn. Motiv..

[24] Leslie Cameron E (2007). Measures of human olfactory perception during pregnancy. Chem. Senses.

[25] Fjaeldstad AW, Nørgaard HJ, Fernandes HM (2019). The impact of acoustic fMRI-noise on olfactory sensitivity and perception. Neuroscience.

[26] Dalton P, Wysocki CJ (1996). The nature and duration of adaptation following long-term odor exposure. Percept. Psychophys..

[27] Doty RL (1978). Intranasal trigeminal stimulation from odorous volatiles: Psychometric responses from anosmic and normal humans. Physiol. Behav..

[28] Hummel T, Roudnitzky N, Kempter W, Laing D (2007). Intranasal trigeminal function in children. Dev. Med. Child Neurol..

[29] Schredl M, Hoffmann L, Sommer JU, Stuck BA (2014). Olfactory stimulation during sleep can reactivate odor-associated images. Chemosens. Percept..

[30] Hidano T, Fukuhara M, Iwawaki S, Spielberger CD (2000). State-Trait Anxiety Inventory form JYZ.

[31] Spielberger CD, Gorsuch RL, Lushene RE (1970). Manual for the State-Trait Anxiety Inventory.

[32] Monk TH (1989). A visual analogue scale technique to measure global vigor and affect. Psychiatry Res..

[33] Agnew HW, Webb WB, Williams RL (1966). The first night effect: an EEG study of sleep. Psychophysiology.

[34] Berry R (2018). The AASM Manual for the Scoring of Sleep and Associated Events: Rules, Terminology and Technical Specifications.

[35] Takeuchi T, Miyasita A, Inugami M, Sasaki Y (1996). Construction of rating scale for dream property (DP scale) and its validation by physiological variables. Jpn. J. Psychol..

[36] Takeuchi T, Ogilvie RD, Ferrelli AV, Murphy TI, Belicki K (2001). The dream property scale: An exploratory English version. Conscious. Cogn..

[37] Faul F, Erdfelder E, Lang A-G, Buchner A (2007). G*Power 3: A flexible statistical power analysis program for the social, behavioral, and biomedical sciences. Behav. Res. Methods.

[38] Faul F, Erdfelder E, Buchner A, Lang A-G (2009). Statistical power analyses using G*Power 3.1: Tests for correlation and regression analyses. Behav. Res. Methods.

[39] JASP Team. JASP (Version 0.17.2) [Computer software]. (2023)

[40] Kass RE, Raftery AE (1995). Bayes factors. J. Am. Stat. Assoc..

[41] Bensafi M (2003). Olfactomotor activity during imagery mimics that during perception. Nat. Neurosci..

[42] Mainland J, Sobel N (2006). The sniff is part of the olfactory percept. Chem. Senses.

[43] Laing DG (1983). Natural sniffing gives optimum odour perception for humans. Perception.

[44] Johnson BN, Mainland JD, Sobel N (2003). Rapid olfactory processing implicates subcortical control of an olfactomotor system. J. Neurophysiol..

[45] LeDoux JE (1993). Emotional memory systems in the brain. Behav. Brain Res..

[46] Blake Y, Terburg D, Balchin R, van Honk J, Solms M (2019). The role of the basolateral amygdala in dreaming. Cortex.

[47] Murata K (2018). [Neural mechanisms of comfortable feeling induced by odors bringing back of memories] Kaori no natsukashisa no shinkei mechanisms (in Japanese). Cosmetology.

[48] Zatorre RJ, Jones-Gotman M, Rouby C (2000). Neural mechanisms involved in odor pleasantness and intensity judgments. NeuroReport.

[49] McCormick DA, Bal T (1994). Sensory gating mechanisms of the thalamus. Curr. Opin. Neurobiol..

[50] de Melo GD (2021). COVID-19–related anosmia is associated with viral persistence and inflammation in human olfactory epithelium and brain infection in hamsters. Sci. Transl. Med..

[51] Karamali K, Elliott M, Hopkins C (2022). COVID-19 related olfactory dysfunction. Curr. Opin. Otolaryngol. Head Neck Surg..

[52] Xydakis MS (2021). Post-viral effects of COVID-19 in the olfactory system and their implications. Lancet Neurol..

[53] Cain WS, Gent JF (1991). Olfactory sensitivity: Reliability, generality, and association with aging. J. Exp. Psychol. Hum. Percept. Perform..

[54] Kareken DA, Mosnik DM, Doty RL, Dzemidzic M, Hutchins GD (2003). Functional anatomy of human odor sensation, discrimination, and identification in health and aging. Neuropsychology.

[55] Stevens JC, Dadarwala AD (1993). Variability of olfactory threshold and its role in assessment of aging. Percept. Psychophys..

[56] de Almeida RN, Motta SC, de Brito Faturi C, Catallani B, Leite JR (2004). Anxiolytic-like effects of rose oil inhalation on the elevated plus-maze test in rats. Pharmacol. Biochem. Behav..

[57] Polonini H (2020). Intranasal use of lavender and fennel decreases salivary cortisol levels and improves quality of sleep: A double-blind randomized clinical trial. Eur. J. Integr. Med..

[58] Miwa T (2019). Clinical practice guidelines for the management of olfactory dysfunction—Secondary publication. Auris Nasus Larynx.

