# Supplementary material for: Subjectively intense odor does not affect dream emotions during rapid eye movement sleep
Source: Sci Rep. 2023 Jun 27;13:10442. doi: 10.1038/s41598-023-37151-8 (PMC10299998; doi:10.1038/s41598-023-37151-8)
Supplement: Supplementary file 1 — Supplementary Tables. [file 41598_2023_37151_MOESM1_ESM.docx]

Subjectively intense odor does not affect dream emotions during rapid eye movement sleep

Supplementary Table S1. Word pairs for odor ratings at screening

| Cheap | Expensive |
| --- | --- |
| Warm | Cold |
| Masculine | Feminine |
| Calm | Activating |
| Weak | Intense |
| Have never smelled | Have smelled |
| Unfamiliar | Familiar |
| Nostalgic | Novel |
| Feel distant from | Feel close to |
| Positive | Negative |
| Unattractive | Attractive |
| Pleasant | Unpleasant |
| Unpreferred | Prefer |

Words were used for a 9-point semantic differential method scale. Words in the left and right columns were assigned 1 and 9 points, respectively. Points 1 and 9 were labeled as completely/extremely, and 5 points (intermediate) were labeled as neither.

Supplementary Table S2. Question and word pairs for the VAS of the pre- and post-sleep conditions

| How is your energy? | | |
| --- | --- | --- |
|  | Lost energy | Full of energy |
| How tired are you? | | |
|  | Not tired | Exhausted |
| How much are you worried about your health? | | |
|  | Not worried | Very much worried |
| How much are you irritated? | | |
|  | Not irritated | Very much irritated |
| How motivated are you? | | |
|  | Less motivated | Fully motivated |
| How anxious are you? | | |
|  | Less anxious | Very much anxious |
| How depressed are you? | | |
|  | Less depressed | Fully depressed |

Words were used to assess the VAS. The left/right columns correspond to both ends of the scale (0 mm/100 mm). VAS, visual analog scale.
